# Supplementary material for: Multi‐dataset identification of innovative feature genes and molecular mechanisms in keratoconus
Source: J Cell Mol Med. 2024 Sep 19;28(18):e70079. doi: 10.1111/jcmm.70079 (PMC11412914; doi:10.1111/jcmm.70079)
Supplement: Supplementary file 2 — Material S2. [file JCMM-28-e70079-s002.zip › 7.GSEA analysis/1.ARL11/KEGG_PORPHYRIN_AND_CHLOROPHYLL_METABOLISM.html]

Details for gene set KEGG\_PORPHYRIN\_AND\_CHLOROPHYLL\_METABOLISM[GSEA]

|  || Dataset | ARL11.group.cls#h\_versus\_l |
| Phenotype | group.cls#h\_versus\_l |
| Upregulated in class | h |
| GeneSet | KEGG\_PORPHYRIN\_AND\_CHLOROPHYLL\_METABOLISM |
| Enrichment Score (ES) | 0.5261859 |
| Normalized Enrichment Score (NES) | 1.7269472 |
| Nominal p-value | 0.00591716 |
| FDR q-value | 0.31502885 |
| FWER p-Value | 0.303 |
Table: GSEA Results Summary

  

Fig 1: Enrichment plot: KEGG\_PORPHYRIN\_AND\_CHLOROPHYLL\_METABOLISM      
 Profile of the Running ES Score & Positions of GeneSet Members on the Rank Ordered List

  

| SYMBOL | TITLE | RANK IN GENE LIST | RANK METRIC SCORE | RUNNING ES | CORE ENRICHMENT || 1 | UGT2B7 | na | 1178 | 0.368 | 0.0787 | Yes |
| 2 | ALAS2 | na | 2163 | 0.285 | 0.1376 | Yes |
| 3 | UGT2B15 | na | 2914 | 0.244 | 0.1908 | Yes |
| 4 | UGT2B11 | na | 2923 | 0.244 | 0.2653 | Yes |
| 5 | CP | na | 3200 | 0.232 | 0.3284 | Yes |
| 6 | UGT2B4 | na | 3576 | 0.216 | 0.3838 | Yes |
| 7 | UGT1A8 | na | 3762 | 0.209 | 0.4425 | Yes |
| 8 | UGT1A6 | na | 4803 | 0.172 | 0.4652 | Yes |
| 9 | UGT2B28 | na | 5683 | 0.146 | 0.4844 | Yes |
| 10 | MMAB | na | 5761 | 0.144 | 0.5262 | Yes |
| 11 | CPOX | na | 9075 | 0.074 | 0.4533 | No |
| 12 | UGT2B10 | na | 9893 | 0.062 | 0.4485 | No |
| 13 | ALAD | na | 10010 | 0.060 | 0.4636 | No |
| 14 | FECH | na | 10499 | 0.054 | 0.4660 | No |
| 15 | PPOX | na | 13212 | 0.025 | 0.3956 | No |
| 16 | UGT2A3 | na | 13634 | 0.022 | 0.3901 | No |
| 17 | HMBS | na | 15338 | 0.009 | 0.3437 | No |
| 18 | UROD | na | 16199 | 0.002 | 0.3196 | No |
| 19 | BLVRB | na | 16550 | -0.000 | 0.3096 | No |
| 20 | GUSB | na | 16664 | -0.001 | 0.3066 | No |
| 21 | FTH1 | na | 16928 | -0.003 | 0.2999 | No |
| 22 | HMOX2 | na | 16987 | -0.003 | 0.2992 | No |
| 23 | BLVRA | na | 20393 | -0.027 | 0.2092 | No |
| 24 | UGT2A1 | na | 20689 | -0.029 | 0.2096 | No |
| 25 | EARS2 | na | 20911 | -0.031 | 0.2125 | No |
| 26 | FTMT | na | 22684 | -0.047 | 0.1759 | No |
| 27 | UROS | na | 23654 | -0.058 | 0.1657 | No |
| 28 | HCCS | na | 25421 | -0.082 | 0.1398 | No |
| 29 | COX15 | na | 25494 | -0.083 | 0.1631 | No |
| 30 | ALAS1 | na | 26487 | -0.098 | 0.1646 | No |
| 31 | COX10 | na | 27120 | -0.111 | 0.1803 | No |
| 32 | HMOX1 | na | 27856 | -0.127 | 0.1981 | No |
Table: GSEA details [plain text format]

  

Fig 2: KEGG\_PORPHYRIN\_AND\_CHLOROPHYLL\_METABOLISM      
 Blue-Pink O' Gram in the Space of the Analyzed GeneSet

  

Fig 3: KEGG\_PORPHYRIN\_AND\_CHLOROPHYLL\_METABOLISM: Random ES distribution      
 Gene set null distribution of ES for **KEGG\_PORPHYRIN\_AND\_CHLOROPHYLL\_METABOLISM**
